# Little but Intense: Using a HIIT-Based Strategy to Improve Mood and Cognitive Functioning in College Students

**DOI:** 10.3390/healthcare11131880

**Published:** 2023-06-29

**Authors:** Inmaculada Concepción Martínez-Díaz, Luis Carrasco Páez

**Affiliations:** 1 BIOFANEX Research Group, Department of Human Movement and Sports Performance, University of Seville, E-41013 Seville, Spain; 2 BIOFANEX Research Group, Department of Physical Education and Sports, University of Seville, E-41013 Seville, Spain; lcarrasco@us.es

**Keywords:** high-intensity exercise, mood states, cognitive functioning, working memory

## Abstract

Looking for useful and motivational strategies for promoting healthy habits and improving cognitive functioning in young populations, the aim of the present study was to determine if a single bout of high-intensity interval exercise could stimulate mood and working memory in college students. A total of 25 male subjects (mean ± SD, age: 21.7 ± 2.1 years; height: 1.77 ± 0.06 m; weight: 72.6 ± 8.4 kg; body mass index: 23.1 ± 1.4 kg/m^2^; VO_2peak_: 47.1 ± 9.3 mL/kg/min) participated voluntarily in this study. Participants underwent a high-intensity interval exercise consisting of 10 × 1 min of cycling at VO_2peak_ power output. The Profile of Mood States (POMS) questionnaire and Digit Span Test (DST) were administered at three assessment time points: (a) pre-intervention assessment, (b) post-intervention assessment, and (c) 30 min post-intervention. The mood states decreased significantly after exercise; however, a significant increase in mood was found after 30 min of recovery. A significant post-exercise increase in DST performance was observed; moreover, DST scores obtained 30 min after exercise remained higher than those assessed pre-exercise. In conclusion, a single bout of HIIT induces acute positive changes in mood states in male college students and seems to be a powerful stimulus for cognitive functioning.

## 1. Introduction

Major global advances in information and communication technologies have generated important effects in terms of productivity and efficiency in a number of sectors. As a result, there have been very detrimental changes in the population’s lifestyles, including, among others, decreases in physical activity, lack of rest, excessive use of screens, and an inadequate diet, assuming, at the same time, a higher prevalence of disorders and diseases.

Focusing only on physical activity, numerous studies have pointed out that a low level of physical exercise practice is linked to a higher risk factor for premature death and chronic diseases (type 2 diabetes, metabolic syndrome, cardiovascular diseases, etc.) [1,2,3,4], as well as a negative impact on mental well-being, in which a high risk of anxiety and depression is included [5,6,7].

In the face of this, opting for a balanced diet and, above all, engaging in regular physical exercise are basic actions that, endorsed by the scientific community, are linked to greater longevity [8,9], lower risk of cardiovascular problems [10,11,12], better quality of life [13], lower risk of diabetes [14,15], better mental health [6,16], and improved cognitive functioning [17,18], among other benefits. However, and although the effects of a healthy lifestyle are well known, numerous studies have warned of the low level of physical exercise practice in adolescents [19,20], who argue that, in addition to laziness and reluctance, the main reason for their inactivity lies in the lack of time as a result of their great dedication to study tasks. Interestingly, these data coincide with studies conducted on university populations [21], in which, in addition to the factors mentioned above, other variables that can affect the level of physical activity are associated with mood, tobacco, and alcohol and drug consumption, as well as the individual perception of whether or not the physical exercise practiced in previous educational stages was a rewarding experience [22].

Considering that there is enough evidence to suggest that young people who participate in vigorous exercise programs gain greater health benefits than those who exercise at light- and moderate-intensity levels, High-Intensity Interval Training (HIIT) seems to be an effective exercise-related approach for this population [23]. Furthermore, HIIT is seen as a time-saving option due to its characteristic of repeated bouts of high-intensity effort interspersed with passive rest or low-intensity exercise [24,25]. Consequently, it is not surprising that HIIT has enjoyed a growing international popularity during the last years, ranking second in fitness trends for 2020 (the top five since 2014) [26].

Previous HIIT studies conducted on adolescents and young people have reported hopeful results regarding cardiorespiratory fitness, body composition, and health-related metabolic adaptations. Nevertheless, there is little information about the impact of HIIT on the mental health and cognitive functioning of young people [23]. A recent meta-analysis revealed that HIIT appears to induce moderate improvements in mental health, depression severity, and perceived stress in nonactive individuals and slight improvements in mental well-being compared to active subjects. Furthermore, HIIT may enhance sleep quality and reduce psychological distress [27]. Nonetheless, it is important to note that active individuals report more positive affective responses (i.e., feelings of pleasure) than sedentary ones when engaging in exercises of moderate to high intensity. Conversely, both insufficiently active and active individuals experienced some pleasure during a few work bouts (i.e., 3–4) of low-volume HIIT, although the affective responses became increasingly unpleasant for insufficiently active individuals over time [28]. In any event, it appears that HIIT sessions performed at a strenuous intensity (especially if they are done under an imbalanced work:recovery ratio, e.g., 1:0.5) may also be detrimental to enjoyment and affective feelings, suggesting that exercise intensity is a critical factor in influencing these responses. Likewise, Oliveira et al. suggested that the greater reliance on anaerobic metabolism during HIIT might be a factor that adversely affects feeling responses and induces mood disturbances [25].

The impact of HIIT on cognitive functioning is still uncertain [29,30]. A recent systematic review revealed that the majority of HIIT interventions had a positive effect on executive functions (EF) and that the beneficial effects on EF related to acute HIIT occurred regardless of exercise type, modality, rest interval, work:recovery time ratio, intensity, sex, and physical fitness level [30]. Kao et al. [31] observed that, after a single session of HIIT and moderate-intensity continuous exercise (MICT), subjects showed improved performance in inhibitory control tasks following HIIT as compared to MICT. However, other studies discovered that adolescents’ response times during these evaluating tasks decreased similarly after both HIIT and MICT interventions in comparison to pre-exercise. On the contrary, other studies have shown opposite results, revealing enhancement or deleterious effects on memory and cognitive functions when using HIIT in different situations [25,32]. In this sense, it is possible to find some publications in the literature identifying intense exercise, not only HIIT, as one of the causes of cognitive impairment and memory loss [33,34].

Considering these discrepancies and the need to have a better understanding about the potential effects of intense exercise on executive functioning, the purpose of this study was to determine the acute effects of a single HIIT session on mood and working memory.

## 2. Materials and Methods

### 2.1. Participants

After applying the inclusion criteria (males students; ages between 18 and 25 years; good general health; and not consuming alcohol, tobacco, and/or drugs) and exclusion criteria (psychological/psychiatric disorders or diseases, bilingualism or multilingualism, musicians, and being under medical treatments with possible effects on the central nervous system), 25 participants (mean ± SD, age: 21.7 ± 2.1 years; height: 1.77 ± 0.06 m; weight: 72.6 ± 8.4 kg; body mass index: 23.1 ± 1.4 kg/m^2^; VO_2peak_: 47.1 ± 9.3 mL/kg/min; physical activity—total IPAQ = 5877.6 ± 1668.2 MET/min/wk) were selected to participate in the present study.

### 2.2. Procedures

Once the sample was selected, and prior to the start of the intervention, the participants were informed, both verbally and in writing, about the characteristics of the research, and all of them signed the corresponding informed consent form. The Ethics Committee of the University of Seville approved the protocol. With a single-group quasi-experimental design, participants had to visit the Sports Science Laboratory of the University of Seville on two occasions, separated by at least 5 days.

In the first visit to the laboratory (session 1), the participants were subjected to a body composition analysis (bioelectrical impedance, TANITA BC-418MA) and graded exercise test (GXT) on a cycloergometer (Ergoline Ergoselect 200) to obtain their VO_2peak_ as the physiological reference. For this purpose, each subject pedaled for 5 min at 50 W (cadence of 70 rpm) with increases of 25 W every minute until exhaustion (i.e., when the subject indicated a score of 19–20 on the Borg scale [35], when the gas exchange ratio exceeded 1.10, when the maximum heart rate -HR_max_- was at 90% of the individually predicted, and/or when the pedaling cadence dropped below 60 rpm for more than 5 s).

During GXT, a gas exchange analysis (CPX, MedGraphics, St Paul, MN, USA) was performed, as well as heart rate monitoring (X-Scribe, Mortara, Milwaukee, WI, USA). The power output (watts) at VO_2peak_ was used in the HIIT session carried out in the second visit to the laboratory (session 2), where the pre-, post-, and 30 min post-intervention assessment time points were established.

### 2.3. Pre-Intervention

This phase began with resting HR monitoring, the recording of the mean blood pressure (MBP) (MBP was calculated from the diastolic and systolic blood pressures (DBP and SBP, respectively) and pulse pressure (PP = SBP − DBP) adjusted by systolic fractions (SF), as suggested by Moran et al. [36] for vigorous physical exercise (50%): MBP = SBP + (PP/2)), and the fulfilling of the Profile of Mood States (POMS) questionnaire [37]. We used the original version that contains 65 items with a 5-point scale for each one (“0 = not at all” to “4 = very strong”) for the period of the “past week, including today” that belonged to following dimensions: depression (DEP), tension–anxiety (T–A), cholera–hostility (C–H), fatigue (FAG), vigor (VIG), and confusion (CFS). Moreover, the POMS Score Index (iPOMS) was calculated using the equation proposed by Fontani et al. (2009): iPOMS = VIG/[(T − A + DEP + C − H + FAG + CFS)/5].

Finally, subjects’ working memory capacity was assessed by means of the Digit Span Test (DST-WAIS IV) [38]. This task consists of increasingly long sequences of random numbers that are orally presented to the participants, who have to repeat the sequence under two conditions: forward (DST-D), in which sequence of digits has to be repeated in the same order as presented, and backward (DST-I), where the digit sequence has to be repeated in reverse order. In both conditions, the task is stopped when a subject fails to recall at least two series of the same length or repeats the last sequence correctly (eight digits for DST-I and nine for DST-D). Thus, the DST performance is defined by three scores: DST-D (nine points maximum), DST-I (eight points maximum), and total DST score (DST-T), which is the sum of the DST-D and DST-I scores.

### 2.4. HIIT-Based Intervention

Considering the reference values obtained in session 1, the participants underwent a high-intensity interval exercise consisting of 10 repetitions of 1 min of pedaling against a constant resistance that was equivalent to the individual power output at VO_2peak_, with 1 min of passive rest between each exercise interval. HR was recorded throughout the exercise intervention, and as the subjects’ safety control, MBP was assessed after the fifth repetition. Similarly, at the end of the minute of effort, the subjects were asked about their subjective perception of effort on a Borg 6–20 scale [35].

After finishing the HIIT bout, the subjects were asked to complete the POMS questionnaire and the DST (POMS-post and DST-post, respectively).

### 2.5. 30 Min Post-Intervention

Once the post-intervention assessment was concluded, participants were asked to remain seated for 30 min in isolation, without any interference. After this time, they were asked to complete the same two questionnaires (POMS 30 min post and DST 30 min post).

### 2.6. Statistical Analysis

Data were expressed as mean ± standard deviation (SD). The Shapiro-Wilk test was used to evaluate the normal distribution of the variables. Considering that the variables did not follow a normal distribution, the Friedman and Wilcoxon nonparametric tests were used to determine the differences between groups between the pre-intervention and post-intervention evaluations. In addition, the effect size was calculated using the *d*-value proposed by Cohen (1988) [39], interpreted as follows: 0.2 was considered a small effect, 0.5 medium, and 0.8 large. Bivariate correlation was also employed using Spearman’s Rho, in which 0.500 was marked as a positive relationship. The confidence interval established was 95% for a *p*-value of <0.05 as the cutoff for statistical significance.

## 3. Results

As expected, all participants were able to complete the HIIT-based intervention. In general, HR showed a gradual increase during the effort. HR_max_ increased from 146.6 ± 11.6 bpm to 177.8 ± 10.9 bpm, whereas the mean HR increased from 126.3 ± 11.5 bpm to 159.5 ± 14.2 bpm. Likewise, the RPE scores increased from 11.9 ± 2.3 to 18.5 ± 1.8 points.

### 3.1. Effects of HIIT Intervention on Mood States

The results of the POMS questionnaire are shown in Table 1, both partially, i.e., for each of the six dimensions and as a global value (iPOMS). The FAG and CFS increased significantly from the pre- to post-exercise intervention, which was coincident with the lowest iPOMS score observed after HIIT. Interestingly, the FAG and CFS showed a significant decrease 30 min post-exercise, reaching values below those assessed before the intervention. On the contrary, but logically, the iPOMS increased significantly 30 min post-HIIT. On the other hand, repeated decreases in T–A were found from pre- to post-exercise and from post-exercise to 30 min after. However, the DEP showed a slight increase at post-intervention but a significant decrease 30 min post-exercise, reaching values below those found before the intervention.

### 3.2. Working Memory

Working memory was evaluated through performance on the DST test. In this sense, there were three results or variables to be considered: direct order (DST-D, with a maximum of 9 points), inverse order (DST-I, with a maximum of 8 points), and the total score, i.e., the sum of the previous results (maximum 17 points). According to the results shown in Table 2, the HIIT intervention provoked an acute and positive effect on working memory, since DST-I and DST-T increased significantly post-exercise. However, only DST-T remained elevated 30 min after exercise.

### 3.3. Relationships between Mood States and Working Memory

A correlation analysis was carried out on the main variables of the study within the same point of analysis of the intervention to subsequently determine the degree of relationships between variables considering the three points of analysis defined in the intervention. Given the distribution of all the variables under analysis, Spearman’s Rho statistic was determined.

Regarding the relationships between POMS and DST, no significant association was obtained in the pre and post situations. However, 30 min after exercise, significant associations were found between some of these variables. Backward DST (DST-I) was related to both the tension–anxiety (T–A, Rho: 0.531, *p* = 0.006) and vigor (VIG, Rho: 0.439, *p* = 0.028) dimensions of POMS at the same assessment time point. Likewise, the total DST score (DST-T) was related to the same POMS dimensions (T–A, Rho: 0.412, *p* = 0.041; VIG, Rho: 0.459, *p* = 0.021) assessed 30 min after intense exercise.

## 4. Discussion

The main aim of the present study was to determine if a single bout of high-intensity interval exercise was able to stimulate mood and cognitive functioning, since HIIT interventions can be a useful strategy for the promotion of healthy habits and the improvement of academic achievements in college students. Considering our results, it seems that HIIT interventions can boost working memory and exert positive effects on mood states of male college students. Nevertheless, it is important to discuss in more detail to what extent intense exercise modulates mood and how these mood changes impact executive functioning.

### 4.1. High-Intensity Exercise and Mood Changes

As is well known, the exercise intensity can play a key role in mood modulation. In fact, the values in some dimensions of the POMS questionnaire decrease as the intensity of exercise is increased, especially above the anaerobic threshold. However, it is important to take into account what other factors, in addition to the intensity of the effort, can be responsible for these modifications in mood. Considering this idea, authors such as Bryan et al. [40], in their transdisciplinary model on voluntary physical exercise, pointed out that some of these key factors are the set of physiological responses that occur with exercise and also the way in which these responses are interpreted by the subject (as is the case in the subjective perception of effort). In the present study, the subjects rated their efforts at the end as “very hard” and “extremely hard”, which correspond to scores on the RPE scale used here above 18 points. It is conceivable that this subjective interpretation of the effort elaborated from the sensations experienced towards the end of the effort (fatigue, pain, etc.) may condition, in general, these decreases in mood and the results of the test. In this sense, in the study by Schlichta et al. [41], in which participants (young adults) were subjected to intense physical exercise (at 80% of their maximum power) until exhaustion under two different conditions (mental fatigue and suppression of emotions), they observed that, in relation to the control group, those who were subjected to some conditions during the test had difficulty with their exercise performance, there being, on the contrary, no statistically significant differences between the two conditions. In the same line, in the study of Van Cutsem [42], he pointed out that the duration and intensity of a physical task seem to be important factors in the decrease of physical performance due to mental fatigue, the latter being subscribed to a greater perceived effort.

As for the residual effects of intense exercise on mood, it should be noted that, taking as a reference the decrease caused at the end of the exercise, the mood returned practically to the values found before the exercise after 30 min of recovery. These data coincide, in part, with those provided by Hall, Ekkekakis, & Petruzzello [43], who recorded a decrease in tension and an increase in the score on the Feeling Scale after the 20 min of recovery after a progressive and incremental effort on a treadmill.

### 4.2. High-Intensy Exercise and Working Memory

After performing the high-intensity physical exercise, there was an improvement in the results obtained in the DST at the end of the test, and these effects persisted slightly 30 min after the end of the exercise. These results were contrary to those obtained in other studies, such as that of Zhu et al. [44], where, in a physical exercise situation very similar to that of our research, the best results in executive functions occurred after the 10-min recovery period and not right at the end of the physical exercise. In any case, as discussed in the previous sections, the improvement in cognitive performance after an intervention with this type of exercise may be conditioned, both by the intensity of the test, as well as by the whole set of physiological and psychological responses [45,46] that physical exercise per se reports in young and healthy subjects.

### 4.3. High-Intensity Exercise, Mood, and Working Memory Interactions

In general, it might be easy to recognize that our mood can influence, to some degree, our performance in different daily activities. In this regard, several investigations have shown that a number of cognitive functions are modulated by mood [47]. Among the executive functions, it has been shown that working memory and, more specifically, performances on specific evaluation tests are affected by negative mood states [48,49]. However, the results of the present study were somewhat uncertain, since, first, relationships under statistical significance were only found 30 min after high-intensity exercise, at which time, their performances on the different DST tests decreased relative to the previous analysis point. In this line, in order to enable physical exercise to be consolidated for lifestyle habits, it is necessary to give special consideration to the previous mental state of the subject before the practice of physical exercise [50], since, as these authors point out, in sedentary participants, conditions of negative mental fatigue prior to exercise produce less positive affective responses during and after exercise, thus making it difficult for the subject to perceive physical exercise as a rewarding experience.

On the other hand, in addition to the effects that high-intensity physical exercise had on mood and executive functions among our participants, it is essential to take into account that these results may be conditioned by numerous factors, for example, lifestyle habits, the number of hours of sleep, the expectations and feelings of each subject for participating in a research study, etc., thus considering what was suggested by Jirout et al. [51], who expressed that the degree of influence exerted by all the factors that make up the triangle of health, cognitive performance, and mood states is still uncertain.

Finally, we should point out that the possible limitations present in our study are centered, above all, on the lack of a control group and on the size and homogeneity of the sample. Considering that all participants were sports sciences students, their physical activity behaviors (they were active subjects) and their expectations and interests in the health-related effects of exercise could have interfered with the results of this study. It is also important to note that the results from our correlation analyses could not be used to determine the causality between changes in mood states and working memory performance. Moreover, using the DST, we only assessed verbal working memory, so we could not know the effects of intense exercise on visuospatial working memory. Lastly, and according to some theories that try to explain the dynamics of mood states, several individual factors, such as affective state, bodily conditions, daily hassles and uplifts, previous experiences, and anticipated events, could influence the magnitude of mood changes during the exercise intervention in our study.

## 5. Conclusions

According to the results obtained in this study, we can state that a single HIIT session generates an acute effect on mood, increasing feelings of fatigue and confusion in healthy young males. However, this effect is transient, since the iPOMS scores increased 30 min after the intense exercise. Similarly, our HIIT session seems to potentiate subjects’ working memory capacity up to 30 min after HIIT. Although the increases in both mood scores and working memory performance were concurrent after HIIT, the lack of a causal relationship did not allow us to clarify if exercise-related mood changes could play a key role in youth cognitive functioning. Future studies (mainly randomized controlled trials) should be focused on the use of intense exercise as a boosting strategy to improve not only physical health but also well-being and cognitive functioning among young people.

## Figures and Tables

**Table 1:** Partial and total scores recorded in the POMS questionnaire at the three time points of session 2. Contrasts (*p* in table) correspond to the Wilcoxon signed-ranks test performed after the application of Friedman’s test (*p* = 0.019 for T–A; *p* = 0.002 for DEP; *p* = 0.629 for C–H; *p* = 0.084 for VIG; *p* < 0.001 for FAG; *p* = 0.166 for CFS; *p* = 0.006 for iPOMS). Effect size (Cohen’s d) is indicated in parentheses. N = 25.

	Pre	Post	30 Min Post	*p* Pre vs. Post	*p* Post vs. 30 Min Post	*p* Pre vs. 30 Min Post
T–A	8.24 ± 5.33	7.83 ± 4.89	6.04 ± 5.30	0.824 (0.088)	0.031 (0.961)	0.036 (0.926)
DEP	4.42 ± 9.96	5.87 ± 10.96	3.36 ± 8.94	0.269 (0.453)	0.006 (1.314)	0.015 (1.109)
C–H	5.04 ± 4.67	4.78 ± 4.29	4.12 ± 4.01	0.420 (0.326)	0.598 (0.211)	0.242 (0.481)
VIG	14.16 ± 6.37	11.91 ± 5.23	12.84 ± 6.37	0.139 (0.620)	0.093 (0.713)	0.785 (0.108)
FAG	4.76 ± 5.25	11.83 ± 6.19	6.16 ± 5.94	<0.001 (2.282)	<0.001 (2.227)	0.120 (0.654)
CFS	5.52 ± 4.78	7.39 ± 5.50	5.08 ± 5.13	0.030 (1.243)	0.021 (1.045)	0.419 (0.328)
iPOMS	3.77 ± 2.99	2.42 ± 2.21	3.59 ± 1.91	0.002 (1.544)	0.009 (1.234)	0.693 (0.158)

**Table 2:** Partial and total results of the DST test at the three intervention analysis points (mean ± SD). DST-D = direct order; DST-I = reverse order; DST-T: total score. Contrasts (*p* in table) correspond to the Wilcoxon signed-ranks test performed after the application of Friedman’s test (*p* = 0.079 for DST-D; *p* = 0.108 for DST-I; *p* = 0.022 for DST-T). The effect size (Cohen’s d) is indicated in parentheses. N = 25.

	Pre	Post	30 Min Post	*p* Pre vs. Post	*p* Post vs. 30 Min Post	*p* Pre vs. 30 Min Post
DST-D	6.04 ± 1.06	6.48 ± 1.31	6.24 ± 2.24	0.234 (0.490)	0.430 (0.320)	0.114 (0.666)
DST-I	4.56 ± 0.92	5.22 ± 1.26	4.80 ± 1.73	0.050 (0.852)	0.695 (0.156)	0.264 (0.458)
DST-T	10.60 ± 1.35	11.70 ± 1.89	11.04 ± 3.76	0.011 (1.189)	0.628 (0.195)	0.042 (0.891)

## Data Availability

The data presented in this study are available on request from the corresponding author. The data are not publicly available due to ethical reasons.
